# The Use of Fitness-Fatigue Models for Sport Performance Modelling: Conceptual Issues and Contributions from Machine-Learning

**DOI:** 10.1186/s40798-022-00426-x

**Published:** 2022-03-03

**Authors:** Frank Imbach, Nicolas Sutton-Charani, Jacky Montmain, Robin Candau, Stéphane Perrey

**Affiliations:** 1 Seenovate, Montpellier, France; 2 DMeM, INRAe, Univ Montpellier, Montpellier, France; 3 Euromov Digital Health in Motion, Univ Montpellier, IMT Mines Alès, Montpellier, France

**Keywords:** Fitness-Fatigue, Machine-learning, Performance, Control theory, Ensemble learning

## Abstract

The emergence of the first Fitness-Fatigue impulse responses models (FFMs) have allowed the sport science community to investigate relationships between the effects of training and performance. In the models, athletic performance is described by first order transfer functions which represent Fitness and Fatigue antagonistic responses to training. On this basis, the mathematical structure allows for a precise determination of optimal sequence of training doses that would enhance the greatest athletic performance, at a given time point. Despite several improvement of FFMs and still being widely used nowadays, their efficiency for describing as well as for predicting a sport performance remains mitigated. The main causes may be attributed to a simplification of physiological processes involved by exercise which the model relies on, as well as a univariate consideration of factors responsible for an athletic performance. In this context, machine-learning perspectives appear to be valuable for sport performance modelling purposes. Weaknesses of FFMs may be surpassed by embedding physiological representation of training effects into non-linear and multivariate learning algorithms. Thus, ensemble learning methods may benefit from a combination of individual responses based on physiological knowledge within supervised machine-learning algorithms for a better prediction of athletic performance.

In conclusion, the machine-learning approach is not an alternative to FFMs, but rather a way to take advantage of models based on physiological assumptions within powerful machine-learning models.

## Key Points

- Fitness-Fatigue models rely on expert knowledge and could be extended to more complex functions, including other factors of athletic performance for prediction purposes while avoiding overfitting.
- Through ensemble learning methods such as stacking, machine-learning approaches are not alternatives to Fitness-Fatigue models but rather a way to improve their predictive capability while preserving expert information in the modelling.

## Introduction

Modelling the effect of training is a major challenge for the sport community since the apparition of the first mathematical models five decades ago [1]. A simplified version of the one from Banister et al*.* [1], the so-called Fitness-Fatigue model (FFM) [2], describes the effect of training on athletic performance relying on some basics of exercise sciences and training theory. It comes with the assumption that each training dose induces two antagonistic responses. One represents little long lasting positive adaptations—the "Fitness"—and the other, large short lasting negative adaptations—the "Fatigue"—that both decay exponentially over time, respectively to their magnitude and rate. Basically, the performance modelled is given by the difference between the fitness and fatigue features. As work proceeded, improvements of the original mathematical structure were developed. Based on more relevant physiological and practical assumptions, models were seeking a better interpretability of parameters and for more accurate predictions. Hence, the underlying impulse responses framework relates to a collection of FFMs [1–9].

Beyond a simple descriptive aim, the main idea behind FFMs was to simulate various training protocols that differ in terms of amount of training (i.e. the "dose") and occurrence in a training process. Through simulations of overload and taper cycles, an optimal training protocol (i.e. appropriate daily workloads and rest time between sessions) that leads to the greatest modelled performance would arise and allow for physiological, practical interpretations and applications.

Using the formalism of transfer functions as a model of relationships between training doses and fitness and fatigue states provides several advantages. First, while Banister et al*.*[1] consider the human performance as the result of the difference between two simple first order transfer functions, the model could be extended to more complex transfer functions. It would allow to model much more sophisticated dynamic relationships between exercise and state variables, according to the complexity and interactions between physiological processes involved in humans [10]. One drawback to this extension would be the loss of direct physiological interpretation, but the model identification phase would not be further complicated. During models training, particular attention regarding overfitting would be paid to ensure generalisation ability on unknown data.

Secondly, transfer functions are the basic tools of control theory [11]. Control theory is a branch of mathematical optimisation that deals with finding a control for a dynamic system over a period of time, such that an objective function is optimised. In classical linear quadratic optimal control problems, the resulting control law (i.e. the training doses here) can be analytically provided from the algebraic representation of the dynamic system and the expected output over a finite temporal horizon. Hence, the optimal control law is a time-varying linear function of the state variables (i.e. fitness and fatigue in our case). Control theory framework thus allows for analytically computing the optimal training doses in a training program, in order to reach a given performance setpoint. Although the first FFMs appeared more than forty years ago, their use to design the optimal training programming is systematically envisaged through simulations, whereas their main advantage relies on their algebraic representation for control purposes.

Finally, the unexploited algebraic structure of FFMs would also provide state observers. By definition, a state observer is a system that provides an estimate of the internal state of a given real system, from measurements of the input and output of the real system [12]. In our field of application, it could be used to precisely estimate the state variables of athletes that are considered in the model (i.e. fitness and fatigue for FFMs) or to adjust the model through performances' observations.

Whilst FFMs are still considered in exercise and sport sciences, they are usually compared to statistical [13, 14] and machine learning forecasting methods [15] for the same purpose. Motivated by their capacity to infer parameters and to predict performances accurately, these possibly alternative methods have the merit of using all available data that stem from any sources (e.g. training-related, environmental, psychological, nutritional). However, when it comes to model a particular athletic performance, there is no consensus on the family of models to apply.

Athletic performance is multi-factorial [13, 16, 17]. Understanding the relationships between training and performance and therefore, to simulate and predict changes in performance is a related complex problem. Hence, the question that arises is what relevance can FFMs ensure in a context of athletic performance modelling? Some potential answers will be discussed in the following sections, by (i) briefly introducing the classical Fitness-Fatigue model and its conceptual issues and (ii) highlighting the contribution of machine-learning methods to the problem.

## Fitness-Fatigue Model and Conceptual Issues

First of all, let us introduce the mathematical structure of FFM. In the original form, fitness and fatigue components are described by first order differential equations. That is, they are convolution based features in which each training input—expressed as a discrete function $\omega \left( i \right)$—is convolved with an exponential transfer function. Hence, the equation of the model is given by the basic level of performance $p^{*}$—a model intercept—and the difference between the two features, with

1 $$\hat{p}\left( n \right) = p^{*} + k_{1} \mathop \sum \limits_{i = 1}^{n - 1} \omega \left( i \right)e^{{ - \frac{1}{{\tau_{1} }}\left( {n - i} \right)}} - k_{2 } \mathop \sum \limits_{i = 1}^{n - 1} \omega \left( i \right)e^{{ - \frac{1}{{\tau_{2} }}\left( {n - i} \right)}} ,\quad n \in {\mathbb{N}}.$$

Here, $\hat{p}\left( n \right)$ is a modelled performance, $k_{1}$ and $k_{2 }$ denote two gain terms and $\tau_{1}$, $\tau_{2}$ denote two time constants for fitness and fatigue impulse responses, respectively. In this form, the model is commonly described as a linear time-invariant system. However, some alternatives motivated by relevant physiological assumptions make the features' parameters varying over time, being dependent on the accumulation of training input [4, 5, 7]. This results in time-variant systems that should better represent the true responses to training.

Yet, the use of FFMs for the purpose of modelling complex phenomena such as athletic performance might be in some ways unsuitable. In the following, we simply decompose the FFMs’ framework into three levels and briefly highlight conceptual issues responsible for errors in prediction.

### The Input: Quantification of Training

The first step of any training effect modelling using FFMs requires to quantify the training itself. Mainly used for modelling the training effects on performance in individual and endurance sports, a few methods for quantifying the training dose exist. Hence, the aforementioned discrete function $\omega \left( i \right)$ can take various expressions. One physiology based on the product of training duration and its exponentially weighted physiological response (e.g. heart rate changes) is termed Training Impulse (TRIMP) [3]. Some other methods commonly rely either on products of volume and intensity parameters, being physiology-based (e.g. using heart-rate variations) [18] or not [19]. When exercise intensity cannot be objectively measured, the session TL is usually estimated using an ex post rating of perceived exertion multiplied by the session's duration [20, 21]. Exercise intensity can also be measured in arbitrary units, especially in cases of technical sport disciplines [22]. On this basis, the training sessions are the only cause of adaptations. That means training responses are independent of any other external factors to training, yet known to impact athletic performance but not accounted for in the model (e.g. environmental factors, nutritional and psychological status). Hence, two identical training sessions that occur at different training stages would induce similar adaptations and responses. Besides, various training sessions (e.g. a low intensity and prolonged exercise, and high intensity and short exercise) may result in similar TL estimates and so Fitness and Fatigue states, despite specific responses and adaptations to exercise exist [23, 24]. For example, two resistance training sessions (a low intensity, high volume and a high intensity, low volume) may lead to similar TL indexes according to the product of exercise volume and intensity [19]. Finally, athletes usually practice endurance and resistance training, and other disciplines to enhance performance.

Since FFMs are sensitive to the nature of the model input [25], a consistent training quantification method that is not biased by the type of training is required across training sessions.

Taking this stand, a univariate configuration of FFMs reduces the space of dimensions around adaptations to training into one single dimension, solely characterised by the training quantification. This is at the expense of all relevant information that can be captured and that may explain a part of athletic performance, even if the training quantification is objectively well estimated.

It also questions training quantification based on arbitrary methods, which might bring "noise" into the modelling in cases where there is an inexact appreciation of the exercise demand by the coach.

### The Function: A Physiological Approximation

Attempting to model athletic performance upon a mathematical representation of physiological principles is obviously commendable. However, it implies being confident in the model itself, leaving no room for vague theoretical approximations. Among the aforementioned variants of the original FFM, improving model complexity (e.g. by adding components in the model) does not guarantee the best model performances [26], even though such models are supposed to represent the physiological responses better. Therefore, the pertinence of adding antagonistic components to the most basic structure (i.e. only based on the fitness component) and more generally, the theoretical hypothesis behind FFMs might be questioned. However, some authors have proposed refinements and extensions of the two-components FFM formulation (see Eq. 1) in light of physiological responses to exercise. On one side, non-linear modifications of the mathematical structure allow model saturation effects [7, 27], describing over-training phenomena. Otherwise, state kinetics were adjusted in order to better represent physiological mechanisms (e.g. tissue remodelling, myosin ATPase activity) [28, 29] through delayed [8], growth and decay kinetics in response to exercise [9]. However, these modifications remain to be more broadly tested in ecological conditions with real data.

### The Output: The Performance

Finally, FFMs attempted to model either an athletic performance during a competitive season, a physical ability that relates to an athletic performance (e.g. mean power or velocity sustained over shorter distances than ones performed during competitions) [5, 30] or a physiological state [31, 32]. In general, choosing the appropriate model output has a strong implication in the modelling process. Modelling changes in physical ability instead of a full discipline-specific performance may allow for repeating less invasive and better controlled testing all along a training process. However, whatever form the output takes (i.e. an athletic performance or a physiological state), its multifaceted nature involves factors that are not considered in the univariate model. Therefore, the training history merely characterised by training loads may only explain a part of changes in the output, somehow resulting in a lack of model performances.

To summarise, FFMs’ ability to predict changes in athletic performance is greatly impacted by univariate modelling issues and a simplification of human physiological adaptations to exercise and training. Moreover, considering only the training loads responsible for changes in athletic performance implies neglect of all related confounding variables that influence both independent and dependent variables, causing spurious associations between input and output of the model.

## A Machine-Learning Perspective of the Problem

Machine-learning models (ML) come with a different approach to the problem. Attempting to predict target variables from sets of co-variables, they foster a multivariate modelling that comprises not only training load variables but all measured variables that may explain changes in athletic performance. Such models are thus considered as valuable solutions for predicting sport performances.

To date, there is a growing attraction of using ML models for modelling adaptations to training [33] as well as for predicting athletic performances [15, 34, 35]. That interest may be justified by a high predictive power of non-linear ML models in many applications, particularly when they are compared to FFMs or other univariate models in sports [15, 35]. Yet, if we compare performances of ML models to FFMs, this is not surprising because the latter represent a restrictive class of models based on strong assumptions (e.g. impulse nature of the response to exercise, the athletic performance resulting from the difference between two states, i.e. fitness and fatigue), which are essentially linear. Such a comparison is also largely biased by the higher degree of freedom of ML models. Therefore, we believe that ML models should not be considered as an alternative to FFMs, but a way to improve and broaden FFM applications instead.

Expert knowledge and strong physiological assumptions that led to the mathematical framework of FFMs represent valuable information that could be used inside ML models. In addition, raw data may also be considered in order to keep the maximum amount of information and thus, advance the athletic performance modelling through an inclusive perspective. Nevertheless, to our knowledge no studies combining FFMs and ML models have been carried out. To be concrete about FFMs, let us suggest some possible alternatives. First, FFMs are mostly univariate except for a very recent study from Piatrikova et al*.*[36] that included well-being indicators as additional model inputs even though it might question the soundness of linearly combining impulse responses to any well-being indicators. Hence, extension of FFMs could be envisaged through state perturbations, or perturbations directly related to the model's ouput. Alternatively, multivariate extensions of FFMs could be made based on linear models’ generalisation, widely used in statistics and ML.

**Fig. 1:**
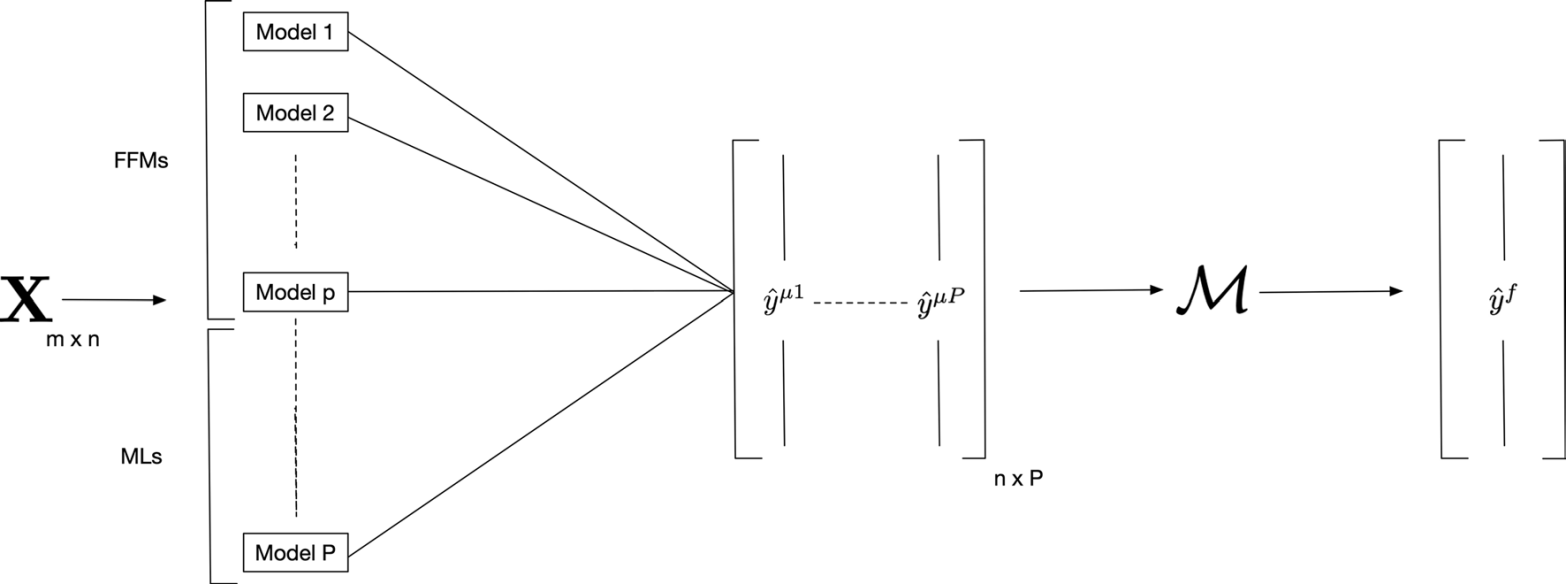
Stacked ensemble learning using several fitness-fatigue and ML models. Briefly, let ${\mathbf{X}}_{m \times n}$ be a first level training data set of $m$ features and *n* observations. Predictions made from the models constitute a prediction matrix (i.e. a second level training data set) of dimension $n \times P$ base-models. Finally, a combiner—or a meta-model—denoted ${\mathcal{M}}$ is trained on these data to predict the final outcome $\hat{y}^{{\text{f}}}$

Since several extensions [3–9] of the former FFM [1] have been developed for predictive applications, there is no consensus about the optimal mathematical structure to be retained. Each of the FFMs variants have their own advantages and drawbacks but they remain close in terms of predictive performances while being heterogeneous in terms of complexity. In addition, predictions made from these models suffer from high bias and low variance, in particular when the target is greatly sensitive to other variables than training load dynamics. For these reasons, combining several FFMs could allow predictive performances to increase. From this perspective, the optimal combination model of predictions computed from different FFMs and ML algorithms could be *learnt* from data as in stacking methods [37] where a regressor is trained to combine several decision tree predictions in an optimal way. Let us consider a set of FFMs predicting athletic performances through fitness and fatigue states, along with ML models that include any other variables of interest, of any kind. Predictions of all base-models (FFMs and ML models) can be aggregated through a "meta-regressor" such as a regularised linear regression. Base-models are composed of various FFMs and ML models which are concurrently trained (within validation procedures such as cross-validation) [38]. The overall process is presented in Fig. 1.

To summarise, the meta-model could thus be used to find the best combination of FFMs and ML models for better prediction purposes [39]. In addition, opting for inherently interpretable "white box" models (i.e. models which provide understandable mappings between inputs and outputs through closed formulas or graphs, such as linear regressions or decision trees) as meta-models [40, 41] could improve experts’ understanding of the FFMs' shortcomings and how ML models can compensate for them. In addition, stacked ensembles do not require a larger sample size than if the models were used separately.

## Conclusion

More than forty years after their initial presentation, FFMs are still used for athletic performance modelling despite mitigated models’ efficiency. Their univariate configuration and a simplification of human physiological adaptations to training may be the leading causes. Yet, it would be worthwhile to extend them to a more sophisticated mathematical framework, still based on bio-physiological knowledge. Sports scientists and coaches would also benefit from their algebraic representation to identify optimal training programming without requiring any simulations. Finally, we truly believe that the ML approach is not an alternative to FFMs, but rather a way to take advantage of models based on control theory. In this sense, ensemble learning should be studied in the specific context of athletic performance modelling, using the actual scientific knowledge within hypothesis-free ML models.

## Data Availability

Not applicable.

## Abbreviations

FFMs: Fitness-Fatigue Models
ML: Machine-learning
